# Separation of Recombination and SOS Response in *Escherichia coli* RecA Suggests LexA Interaction Sites

**DOI:** 10.1371/journal.pgen.1002244

**Published:** 2011-09-01

**Authors:** Anbu K. Adikesavan, Panagiotis Katsonis, David C. Marciano, Rhonald Lua, Christophe Herman, Olivier Lichtarge

**Affiliations:** Department of Molecular and Human Genetics, Baylor College of Medicine, Houston, Texas, United States of America; Uppsala University, Sweden

## Abstract

RecA plays a key role in homologous recombination, the induction of the DNA damage response through LexA cleavage and the activity of error-prone polymerase in *Escherichia coli*. RecA interacts with multiple partners to achieve this pleiotropic role, but the structural location and sequence determinants involved in these multiple interactions remain mostly unknown. Here, in a first application to prokaryotes, Evolutionary Trace (ET) analysis identifies clusters of evolutionarily important surface amino acids involved in RecA functions. Some of these clusters match the known ATP binding, DNA binding, and RecA-RecA homo-dimerization sites, but others are novel. Mutation analysis at these sites disrupted either recombination or LexA cleavage. This highlights distinct functional sites specific for recombination and DNA damage response induction. Finally, our analysis reveals a composite site for LexA binding and cleavage, which is formed only on the active RecA filament. These new sites can provide new drug targets to modulate one or more RecA functions, with the potential to address the problem of evolution of antibiotic resistance at its root.

## Author Summary

In eubacteria, genome integrity is in large part orchestrated by RecA, which directly participates in recombination, induction of DNA damage response through LexA repressor cleavage and error-prone DNA synthesis. Yet, most of the interaction sites necessary for these vital processes are largely unknown. By comparing divergences among RecA sequences and computing putative functional regions, we discovered four functional sites of RecA. Targeted point-mutations were then tested for both recombination and DNA damage induction and reveal distinct RecA functions at each one of these sites. In particular, one new set of mutants is deficient in promoting LexA cleavage and yet maintains the ability to induce the DNA damage response. These results reveal specific amino acid determinants of the RecA–LexA interaction and suggest that LexA binds RecA*_i_* and RecA*_i+6_* at a composite site on the RecA filament, which could explain the role of the active filament during LexA cleavage.

## Introduction

Genetic material is under constant environmental assault. The bacterial recombinase protein RecA is pivotal to DNA repair [1]–[4] and to orchestrate the bacterial DNA damage response (SOS response) against natural, or drug-induced, genotoxic conditions. It is part of an ancient and evolutionarily widespread protein family and, except for a few endosymbionts [5], homologs carry out related functions in archaea [6] and eukaryotes [7], and in some cases mutants are linked to human cancers [8], [9].

To perform its many roles, RecA interacts with multiple partners in *E. coli* [3]. It normally exists in an inactive conformation without bound DNA [10], [11]. Upon DNA damage, an essential first step is the RecA polymerization around a single stranded DNA (ssDNA) in an ATP-dependent fashion [12]–[14]. In this active filament form, it can direct homologous recombination [15], bind to DinI [16], [17] and RecX [18]–[20] to control filament growth [21], [22], and bind the RecFOR complex to repair ssDNA breaks [23]–[25]. RecA is also a co-protease that promotes cleavage of the transcriptional repressor LexA [26] to trigger the expression of over 40 SOS response genes [27]. It also promotes cleavage of UmuD [28]–[31] to become a constituent of the error-prone DNA polymerase V (pol V) [32], [33], in addition to direct interaction with pol V for its activity [33]. Alternately it also interacts with another Y family of DNA polymerase, DinB (pol IV), to directly modulate its mutagenic potential in the translesion DNA synthesis [34]–[36]. It also promotes cleavage of the phage repressor, λCI, triggering induction of the lytic cycle [37], [38]. Every one of these interactions is a potential target to design drugs or mutants that dissect the molecular basis of RecA-dependent genomic repair and stability.

**Figure 1:**
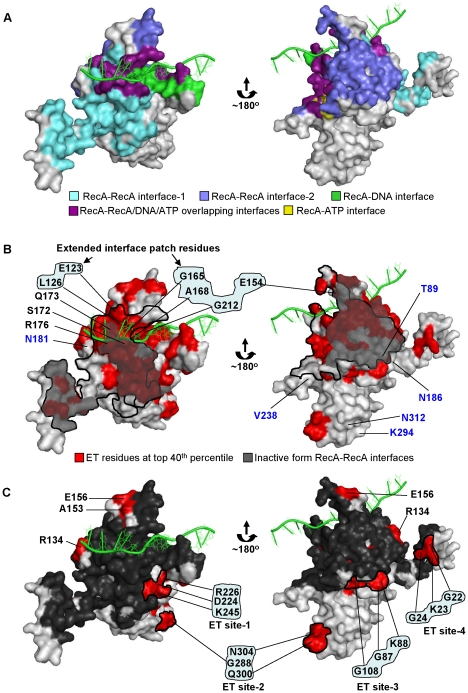
Evolutionary Trace analysis identified clusters of important residues in *E. coli* RecA. (A) The active RecA monomer (PDB:3cmw) showing known structural interfaces. The bound DNA is shown as green cartooned structure. The crystal structures shown in right and left panels are two opposite sides of the RecA monomer. The relative importance of the residues in *E. coli* RecA was computed by Evolutionary Trace analysis of its 201 protein homologs of bacterial origin. (B) The residues ranked in the top 40^th^ percentile of evolutionary importance are highlighted in red color on the active RecA monomer (PDB:3cmw). The RecA-RecA interfaces formed in the active form were contoured with thick lines with the same interface deduced from the inactive monomer structure shown superimposed with grey shade. For clarity, only one of the RecA-RecA interfaces is shown contoured with a thick line, in each side of the monomer. The top-ranked ET residues adjacent to the RecA-RecA interface-1 in the inactive form forming the extended interface patch are highlighted. The control residues of bottom-ET ranked are shown in blue letters. (C) Known structural interfaces are shaded dark grey on the active RecA monomer. The ET clusters (ET site-1,-2,-3, and -4) consisting of 3 or more residues, forming structurally and functionally unknown sites are shown with the residues targeted for site-directed mutagenesis. Note that the ET clusters (shaded red) constituting less than 2 residues or previously characterized residues (E156, A153) though not part of known interfaces, were not included for mutational analyses. The figures representing RecA crystal structures were generated using PyMOL.

There are many crystallographic structures of RecA, or homologs, but most do not include bound DNA, and so are thought to represent the inactive conformation [39]–[50]. More recently, the crystal structure of *E. coli* RecA bound to DNA in the active conformation was solved (hereafter PDB:3cmx) [51]. It showed the ATP binding site, the DNA binding site and RecA-RecA interfaces in a likely active form (Figure 1A). Still, the interaction sites for other partners (such as DinI, RecX, RecFOR, LexA, UmuD, UmuD_2_C, DinB and λCI, as mentioned above) remain unknown. Separately, several mutational studies sought to identify residue determinants of diverse RecA functions, but without yet producing a fully coherent view [52].

To investigate the biological roles of known structural sites and to discover other RecA functional sites, we turned to the Evolutionary Trace (ET). This phylogenomic method [53]–[55] ranks a protein's residues by relative evolutionary importance. A structural map of the top-ranked residues then reveals clusters that indicate active sites and binding sites on the protein surface and that efficiently guide site-directed mutations that block, separate, or rewire functions in eukaryotic proteins [56]–[68]. ET analysis revealed many clusters of top-ranked residues on the *E. coli* RecA surface, which were targeted for mutagenesis followed by functional analysis. This extended and confirmed the biological role of the interfaces revealed in the inactive and active filament structure [51] and, critically, revealed new sites in other regions where mutations separated recombinase activity from co-protease activity for LexA cleavage. Two structurally distant amino acids (G108 and G22) are linked to the RecA-LexA interaction, and their location on RecA subunits *i* and *i*+6 apart in the helical active filament, across the groove, suggests a constraint on a low-resolution, illustrative model of the LexA-RecA interaction.

## Results

### Evolutionary Trace (ET) analysis identified clusters of residues in RecA

In order to identify novel, biologically relevant functional sites in the *E. coli* RecA protein, ET analysis was performed on 201 RecA homologs of bacterial origin. Each residue sequence position was ranked by ET based on how well its variations among homologs correlated with phylogenetic divergences (Figures S1 and S2) [56], [69]. Residue positions ranked in the top 40^th^ percentile rank (thereafter ET_40_) were mapped onto the monomer of the RecA crystal structure, in the active form [51] (Figure 1B, shaded red and maroon). ET_40_ residues formed statistically significant clusters, with a *z*-score of 1.9, and suggested a number of functional surfaces, including as expected known sites such as the RecA-ATP interface, RecA-DNA interface and the two RecA-RecA interfaces (Figure 1A and contoured with a thick line in Figure 1B).

One area of interest includes a cluster of ET_40_ residues that borders the RecA-RecA interface in the inactive structure (residues highlighted in cyan in Figure 1B) but within the RecA-RecA, RecA-DNA interfaces in the active structure (Figure 1A and 1B). It includes residues E123, E154, L126, G212, G165 and A168. The structural data and the ET rank of these residues suggest they may be functionally important for oligomerization, although no experimental evidence has indicated such a role. It is likewise for residues S172, R176 and Q173, which are within the RecA-RecA structural interface common to both active and inactive structures (residue positions shown in Figure 1B). All of these residues were therefore grouped together as the extended RecA-RecA/DNA *interface patch* and chosen for mutational and functional analysis, described below.

Besides this interface patch, other ET_40_ residues formed various other clusters elsewhere on the RecA structure. These were named, arbitrarily, ET site-1 (D224, R226 and K245), ET site-2 (G288, Q300 and N304), ET site-3 (G87, K88 and G108) and ET site-4 (G22, K23 and G24) (residue positions shown in Figure 1C). Since each of these sites suggest a new putative structural interface without any known function, site-specific mutagenesis was performed to probe their function. For all site-directed mutagenesis, amino acids were individually mutated to alanine unless the alanine substitution already existed in a member of the ET sequence dataset. For such exceptions, tyrosine, tryptophan or glycine residues were used depending on their absence from the substitution profile of the targeted position. All mutations were constructed on a low-copy plasmid-borne *recA* gene and transformed into a Δ*recA E. coli* strain [70]. The mutant RecA strains were tested for their UV sensitivity to assess the global impact on RecA function. Representative mutant strains from each ET clusters were also tested for their sensitivity to mitomycin C to demonstrate that the survival phenotypes of these mutants were not specific to UV induced DNA damage (Figure S3). Then, to pinpoint the molecular basis of UV sensitivity, both a P1 recombination assay and a LexA western blot assay were performed to probe the recombinase activity and the induction of LexA cleavage of each RecA variant *in vivo*, respectively.

**Figure 2:**
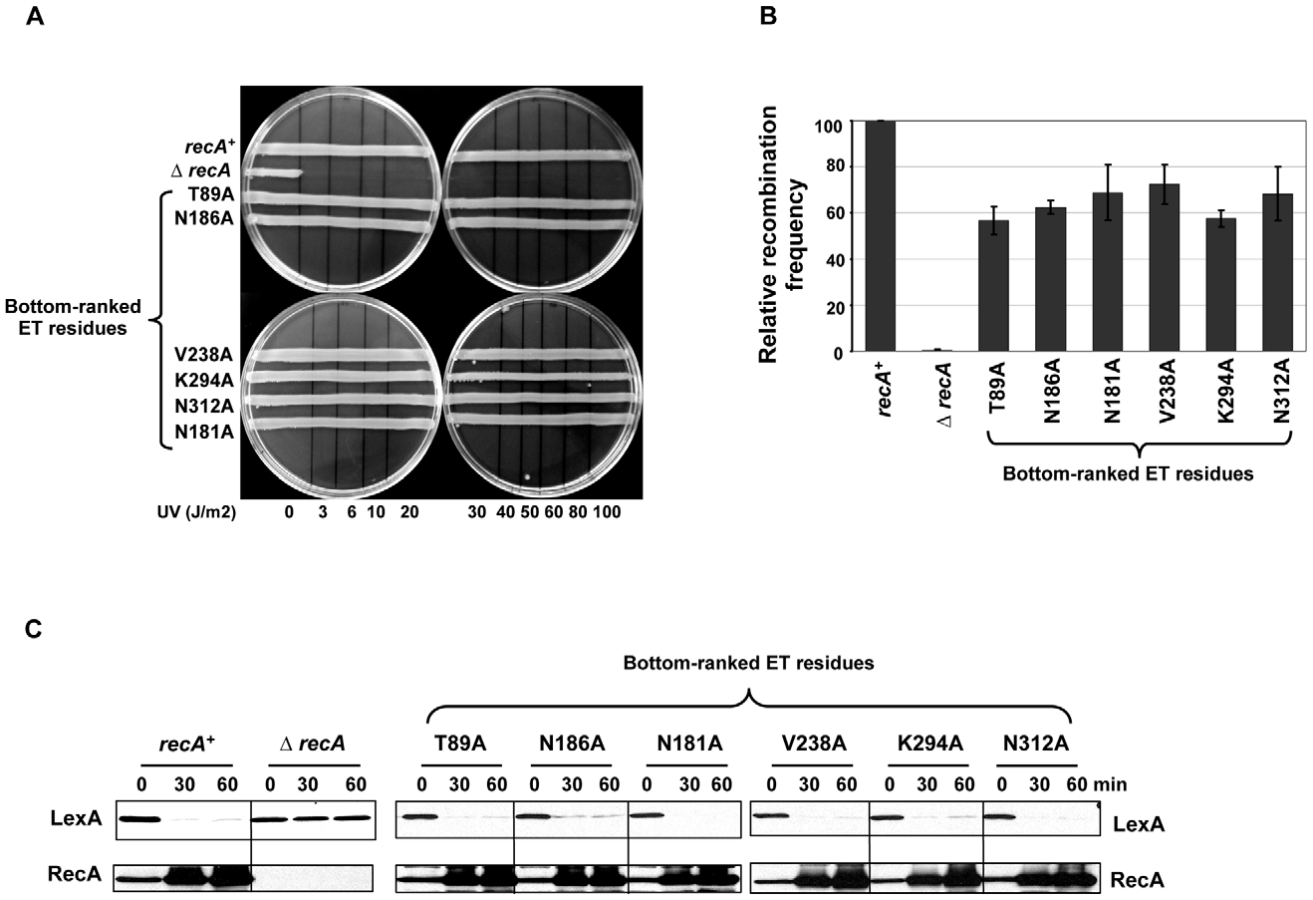
RecA functional assays with control mutants. The effect of RecA mutations in the bottom-ranked ET residues were compared with wild-type *recA* or Δ*recA* in functional assays that tested RecA activity. (A) UV survival assay. LB agar plates showing strains carrying mutations in the bottom-ranked ET residues having no impact on RecA function and survived UV damage like wild-type strain, whereas the Δ*recA* strain could not survive even a very low UV dose (3 Joules/m^2^). The results shown are the representative of three independent assays. (B) P-1 transduction assay. The efficiency of RecA variants to recombine the selectable genetic marker was expressed relative to that of wild-type *recA* strain. All the bottom-ranked ET residue mutant strains had relatively intact recombination efficiencies that ranged from 56 to 72% compared to wild-type *recA* strain. The recombination frequency of wild-type *recA* strain in this case was (4.1±0.6)×10^−5^ per P1 plaque-forming unit. Recombination frequency is corrected for the viability of recipient strains. The relative recombination frequencies are calculated as mean ± S.E. from three independent experiments. (C) *In vivo* LexA cleavage induction analysis by western blot. The mid-log phase cultures of the RecA-WT or mutant strains or the empty vector control were treated with the DNA damaging agent, nalidixic acid (100 µg/mL). Culture aliquots were made at 0 (no treatment), 30, and 60 minutes intervals. Total protein lysates were made and 50 µg of the above fractions were resolved on SDS-PAGE and immunoblotted with anti-LexA antibody. The blots were stripped and re-probed with anti-RecA antibody. LexA cleavage fragments could not be shown as they were highly unstable. Except Δ*recA* strain, all bottom-ranked ET residue mutant strains were equally capable of inducing LexA cleavage similar to wild-type *recA* strain. RecA upregulation is noticed when LexA derepression occurs due to its cleavage in wild-type RecA. The mutant RecA proteins were stable as shown by intact, undegraded RecA protein seen in western blots. All the western analyses were independently carried out at least 3 times and the representative result is shown.

Finally, to validate our ET analysis on RecA, several poorly-ranked ET residues located on the RecA surface (in the worst quartile of importance) were analyzed through site-directed mutagenesis and functional analysis as described above. Such bottom-ranked residues near the known RecA interfaces included T89, N181, N186 and V238, and others that were away from any known RecA interfaces included K294 and N312 (Figure 1B, shown in blue letters). As expected, mutation of these residues displayed no UV sensitivity (Figure 2A), had relatively intact recombination efficiencies that ranged from 56 to 72% compared to wild-type RecA strain as judged by P1 recombination assay (Figure 2B) and were all fully capable of inducing LexA cleavage leading to upregulation of RecA protein (Figure 2C).

### Functional validation of the extended interface patch in RecA active filament

**Figure 3:**
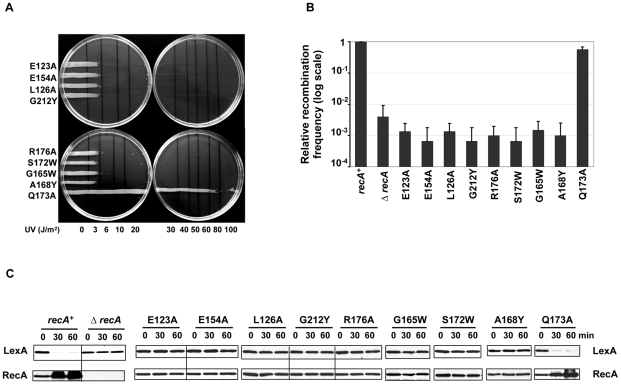
RecA extended interface patch residues are involved in RecA active filament formation. (A) UV survival assay. 8 out of 9 mutations targeting residues in the ET dimer patch were sensitive to UV damage even at low doses (3–6 J/m^2^). All of the 8 UV sensitive mutants showed disrupted recombination and LexA cleavage efficiencies in the P1 transduction assay (B) and in the western analysis of LexA (C) respectively. RecA upregulation was not observed in these UV sensitive mutants upon DNA damage (C). The relative recombination frequencies of RecA mutants (B) are shown in log scale.

In order to test the functional role of the extended interface patch residues (Figure 1B), site-directed mutagenesis was performed. As expected from interference with RecA-RecA or RecA-DNA interactions, either of which would disrupt the basic ability of RecA to form active nucleoprotein filament, these RecA mutant strains within the interface patch were strongly sensitive to very low doses of UV damage (Figure 3A) similar to the empty vector in a Δ*recA* background (Figure 2A) with the exception of the Q173A substitution. As a positive control, the *E. coli* strain with wild-type RecA overcame UV damage (Figure 2A).

To characterize the recombination efficiency, a P1 transduction assay was performed. All variants, except for Q173A, are disrupted for recombination similarly to the Δ*recA* strain (Figure 3B). Likewise, these variants, significantly hindered RecA's ability to promote LexA cleavage upon DNA damage and subsequently failed to up-regulate RecA (Figure 3C). The observation that Q173A mutation showed no effect on RecA activity could be attributed to the lesser importance of this residue, which has the worst rank of the ET_40_ residues in this patch (30th percentile-rank).

Taken together, these mutations confirmed that top-ranked ET_40_ residues in this extended interface patch impair both the recombination and co-protease activities of RecA, and thus are crucial for UV survival. This is consistent with the structural data on the active RecA filament [51]. These residues are directly involved in RecA-RecA and RecA-DNA interaction, and their mutations are thus likely to interfere with the basic assembly or working of the nucleoprotein RecA-DNA filament.

### ET site-1 may be involved in RecA–RecA interaction

**Figure 4:**
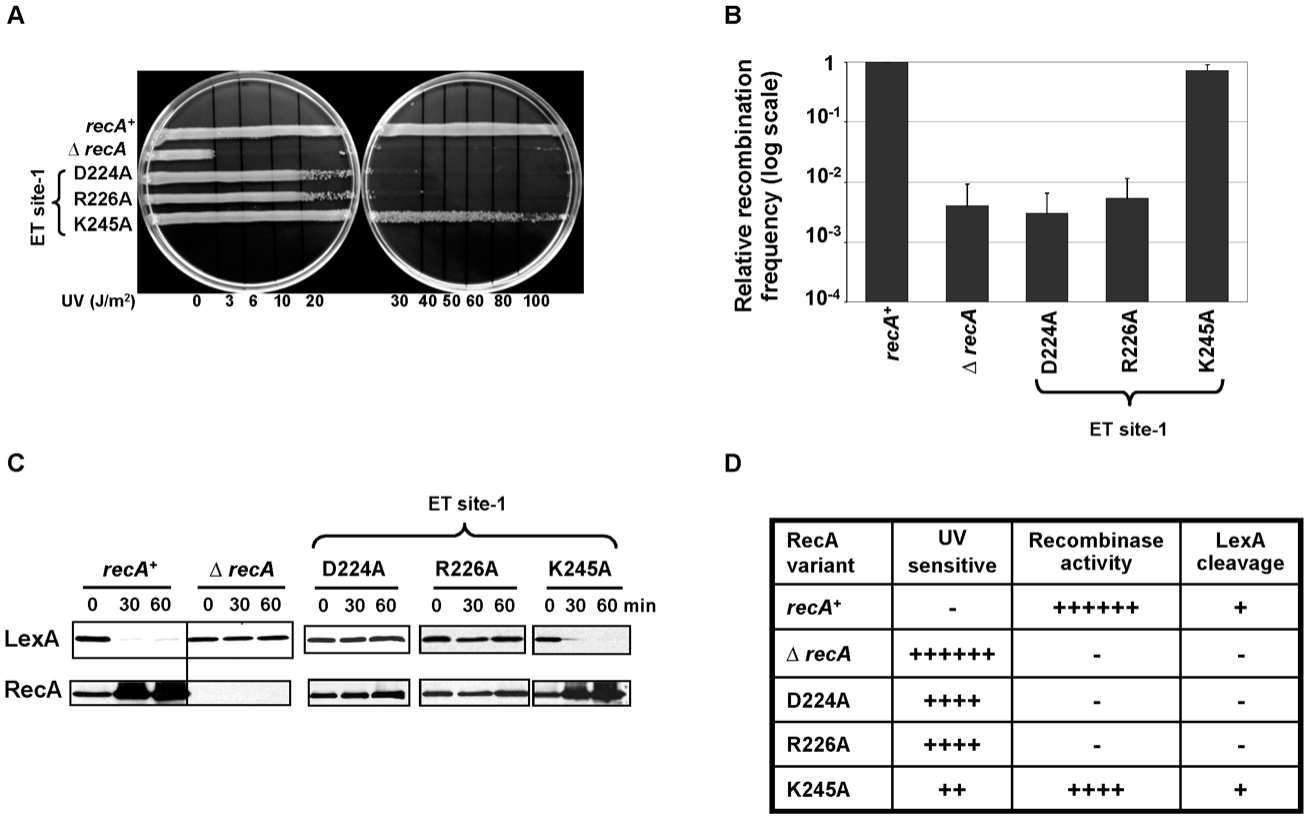
ET site-1 is essential for RecA–RecA homodimerization. LB agar plates showing RecA variants, D224A and R226A sensitive (20 J/m^2^) to UV induced DNA damage in the UV survival assay (A). These variants were also deficient in recombinase activity (B), LexA cleavage induction and RecA upregulation (C). The relative recombination frequencies of RecA mutants (B) are shown in log scale. All the three assays were carried out at least 3 times independently, and the representative figures or data representing the mean ± S.E. are shown. (D) Summary of the mutant strains phenotypes. Under UV sensitivity, 6+ is roughly equivalent to sensitivity at 3 J/m^2^. Under recombinase activity, recombination frequencies equivalent to 100% are indicated by 6+. Under LexA cleavage, if the strains induce LexA cleavage irrespective of the cleavage rate, it is represented as + and if the strains could not induce LexA cleavage at 60 minutes after treatment, it is represented as −.

Two of three ET site-1 RecA variants (R226A and D224A) are UV-sensitive (Figure 1C and Figure 4A). These mutations strongly disrupt recombinase activity of RecA (Figure 4B) and LexA self-cleavage (Figure 4C), similarly to the extended interface patch variants. Assuming that RecA folding is not affected, and given the structural contiguity to the RecA-RecA interface-1 (see Figure 1A and 1C) one reason may be some involvement in RecA-RecA interaction and filament formation. Another possibility is that ET site-1 could play a role in binding to proteins such as RecX, DinI and RecFOR that modulate RecA function.

### ET site-2 specifically affects the recombination function of RecA

**Figure 5:**
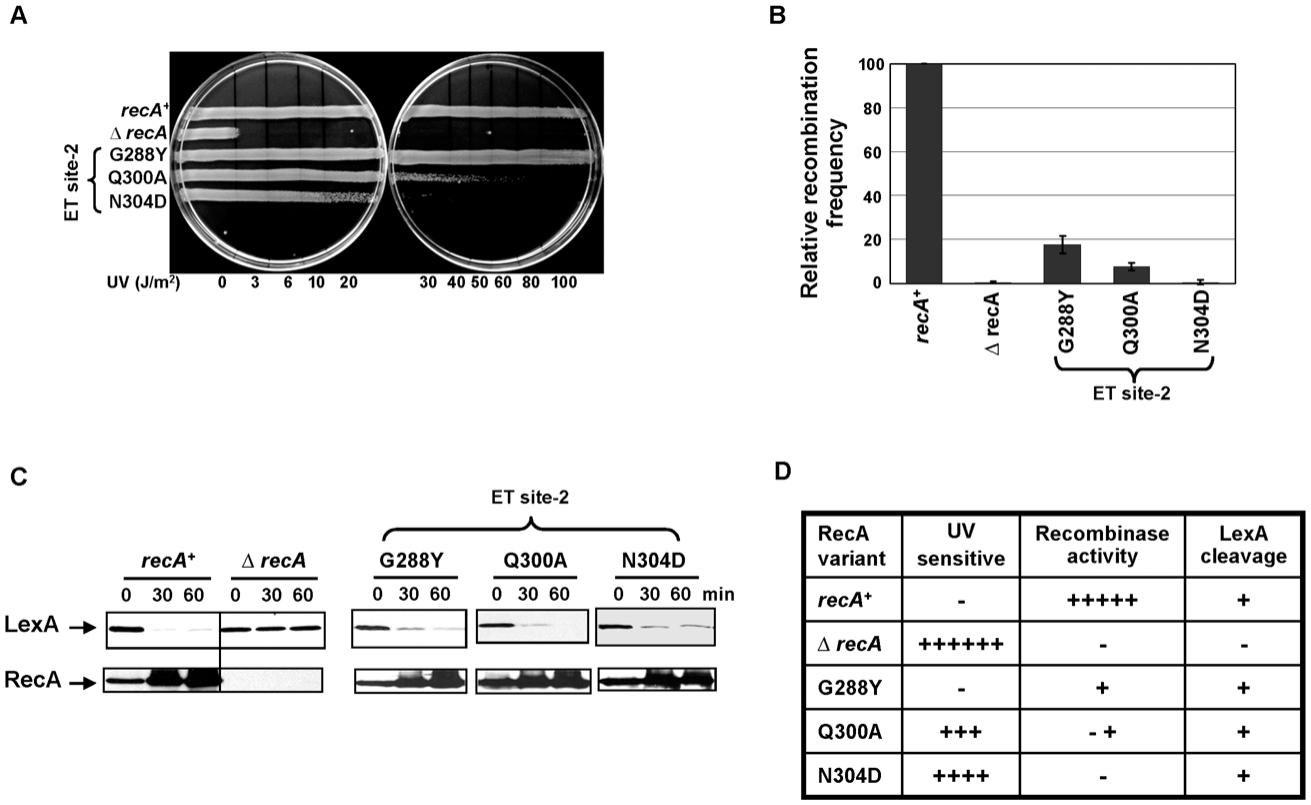
ET site-2 specifically affects the recombination function of RecA. (A) UV survival assay showing RecA variants N304D and Q300A sensitive to UV damage except G288Y. All the 3 variants showed reduced recombinase activity in P1 transduction (B) with N304D mutant strain showing complete disruption similar to Δ*recA* strain, whereas all the three mutant strains induced LexA cleavage and consequent RecA upregulation seen in western analysis (C). All the three assays were carried out at least 3 times independently, and the representative figures or data representing the mean ± S.E. are shown. (D) Summary of the mutant strains phenotypes. Under UV sensitivity, 6+ is roughly equivalent to sensitivity at 3 J/m^2^. Under recombinase activity, recombination frequencies equivalent to 100% are indicated by 5+ ; 1+ equivalent to 20%; −+ indicated roughly 5–10% relative recombination frequency. Under LexA cleavage, strains that induced LexA cleavage irrespective of the rate, was represented as + and those that could not induce LexA cleavage at 60 minutes after treatment, was represented as −.

Mutational analysis of ET site-2 residues (Figure 1C) showed separation of RecA function. First, two of the three mutant strains have abnormal UV sensitivity (Figure 5A). The N304D variant was the most sensitive, followed by Q300A. The G288Y variant displayed no UV sensitivity. Next, all three mutant strains had reduced recombination efficiency (Figure 5B), with the N304D variant being as deficient as the Δ*recA* strain. Finally, LexA cleavage upon DNA damage was intact (Figure 5C), suggesting that the RecA folding and active filament formation required for SOS induction were unaffected. Thus, overall, all these ET site-2 mutations have more severe impact on the recombinase activity than on the SOS response. The N304D mutant, which is completely defective for recombinase activity, displays the clearest example of separation of function. Therefore, these data suggest that the ET site-2 is essential for the recombinase function of RecA. One explanation is that this site may bind to the dsDNA or to other partners essential for recombination events.

### Residues in ET site-3 and site-4 specifically affect LexA cleavage

**Figure 6:**
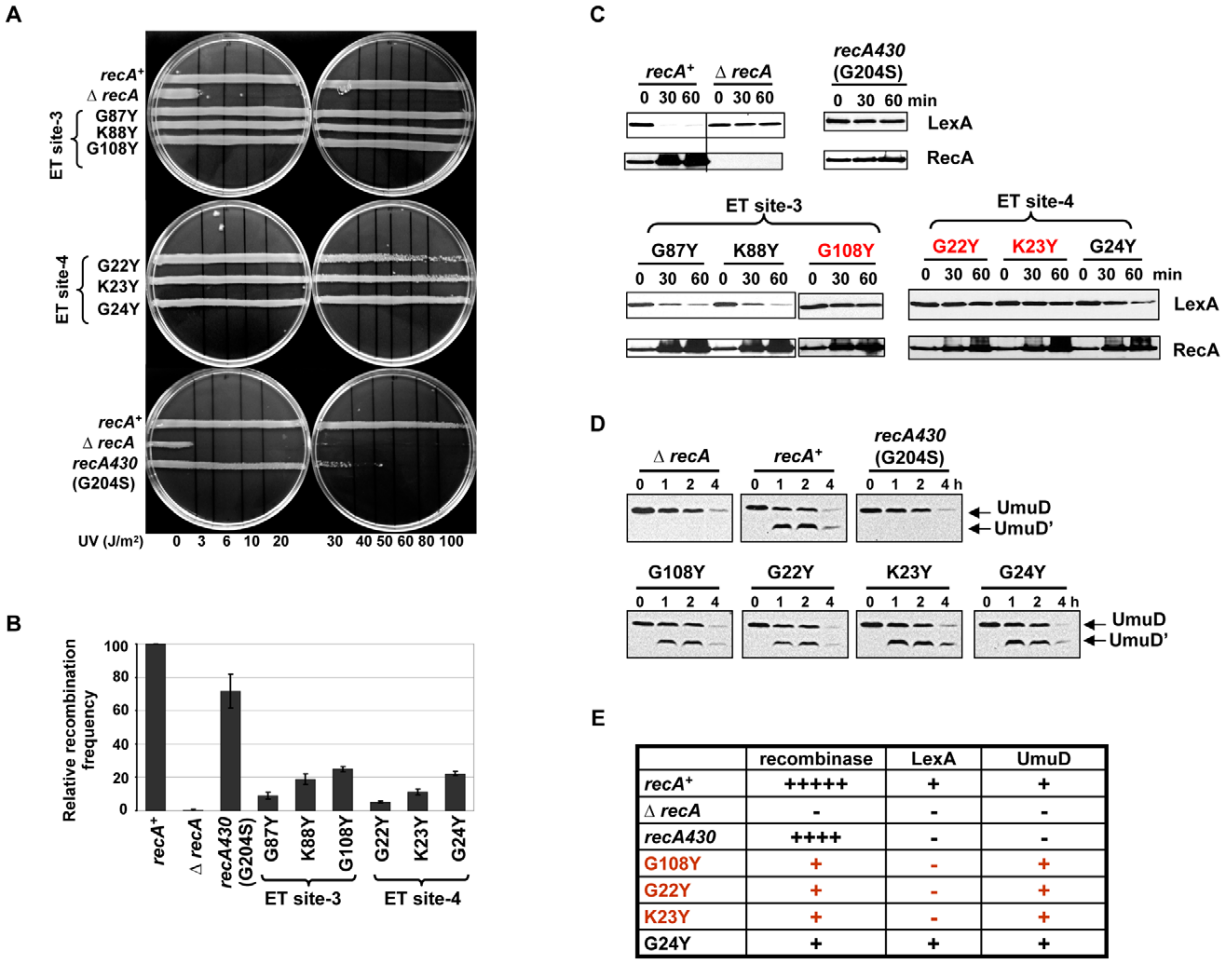
Residues in ET site-3 and site-4 specifically affect LexA cleavage. (A) UV survival assays showing ET site-3 and site-4 residues mutant strains resistant to UV damage except G22Y at higher UV dose (80–100 J/m^2^) and the SOS-deficient RecA variant *recA430* (G204S) sensitive at 30–40 J/m^2^. (B) P1 transduction assay. The recombination efficiency was reduced to 9 to 25% for RecA variants in ET site-3 and 5 to 22% for RecA variants in ET site-4, but *recA430* variant had up to 75% of relative efficiency to recombine. (C) Western analysis of LexA cleavage. RecA variants G87Y and K88Y (ET site-3) and G24Y (ET site-4) induced LexA cleavage similar to RecA-WT. LexA was not cleaved in G108Y (ET site-3) and G22Y and K23Y (ET site-4) variants, but up-regulation of RecA up to 3.5 to 4.6-folds was noticed in these variants. LexA cleavage and RecA upregulation was not seen in *recA430* variant. (D) Western analysis of UmuD cleavage. The *recA*-WT, Δ*recA* and mutant *recA* plasmids were transformed into a LexA cleavage deficient *E. coli* strain. The LexA-repressed UmuD protein was constitutively up-regulated in these strains in the absence of DNA damage at 0 time point. UmuD cleavage to UmuD' is seen in the RecA variants G108Y, G22Y, K23Y and G24Y but not in *recA430* and Δ*recA* strain. Unlike LexA cleavage analysis, UmuD cleavage induction being a late response was assessed at relatively later time points (1, 2 and 4 hours after treatment). In all these assays, *recA* and Δ*recA* represents the Δ*recA* strain carrying either wild-type *recA* or empty vector respectively. All of the above assays were carried out at least 3 times independently, and the representative figures or data representing the mean ± S.E. are shown. (E) Summary of the phenotypes observed for RecA variants. rec- recombinase activity; LexA- induction of LexA autoproteolysis; UmuD- induction of UmuD autoproteolysis.

Mutagenesis of ET site-3 (G87, K88 and G108) and ET site-4 (G22, K23 and G24) (Figure 1C) also produced partial separation of function. In contrast to mutations affecting ET site-2 residues, these variants displayed no UV sensitivity (Figure 6A), except for the G22Y variant, which only becomes UV sensitive at higher doses. All variants displayed lower recombination efficiency compared to wild-type RecA but none as complete as the Δ*recA* strain. The efficiency was down approximately to 9, 19 and 25% for G87Y, K88Y and G108Y in ET site-3 and to 5, 11 and 22% for G22Y, K23Y and G24Y, respectively, in ET site-4 (Figure 6B). Among these residues, the G108Y (in ET site-3), G22Y and K23Y (in ET site-4) showed strongly reduced LexA proteolysis upon DNA damage (Figure 6C, highlighted in red). Strikingly, these three mutant strains exhibited 3.5 to 4.6-fold upregulation of RecA levels, consistent with SOS induction, even in the absence of LexA cleavage after DNA damage. These variants show some similarity to a well-known *recA* mutant, *recA430* (corresponding to G204S) [71]–[73], which is only slightly affected for recombination but deficient for LexA cleavage. In our assays, this variant showed an increase in UV-sensitivity (Figure 6A, lowermost panel), with relatively intact recombination activity, and no ability to induce LexA cleavage. However, this variant did not up-regulate RecA, unlike the G108Y, G22Y and K23Y mutant strains (Figure 6C). Thus RecA upregulation without LexA cleavage upon DNA damage is unique to our three mutant strains. Finally we asked whether this defect in co-protease activity was specific to LexA by testing another substrate of RecA, UmuD, which is also activated upon LexA cleavage. In this case, the active RecA filament mediates UmuD autoproteolysis yielding UmuD'. Since UmuD cleavage induction is a later SOS response than that of LexA, it was analyzed at later time points. Upon DNA damage, we observed an upregulation of UmuD levels in G108Y mutant strain and also a slight upregulation in G22Y and K23Y mutants respectively (data not shown). To analyze UmuD cleavage, we used a *lexA* (def) *E. coli* strain which is constitutive for UmuD expression. Formation of UmuD', the cleavage product of UmuD was visible in G108Y and G22Y mutant strains indicative of self-proteolysis of UmuD induced by RecA (Figure 6D). However in the G24Y mutant, that was shown to cause a slow LexA cleavage, there was a robust upregulation of UmuD like wild-type RecA (data not shown). In addition, the *recA430* mutant strain, in our assay, could not cleave UmuD as well (Figure 6D). The upregulation of UmuD and its subsequent cleavage to UmuD' in G108Y, G22Y and to some extent in K23Y, strengthens the possibility that these variants alter most likely RecA-LexA interaction rather than affecting the overall co-protease activity of RecA.

### Role of G108 and G22 residues in initiating LexA cleavage

The unexpected upregulation of RecA without LexA cleavage after DNA damage could suggest that LexA is sequestered by active RecA filaments, leading to SOS induction. Specifically, mutation of just one of the two residues, G108 or G22, could leave the ET-site with the other residue intact and able to bind LexA to RecA, effectively titrating it away from transcriptional repression irrespective of cleavage.

**Figure 7:**
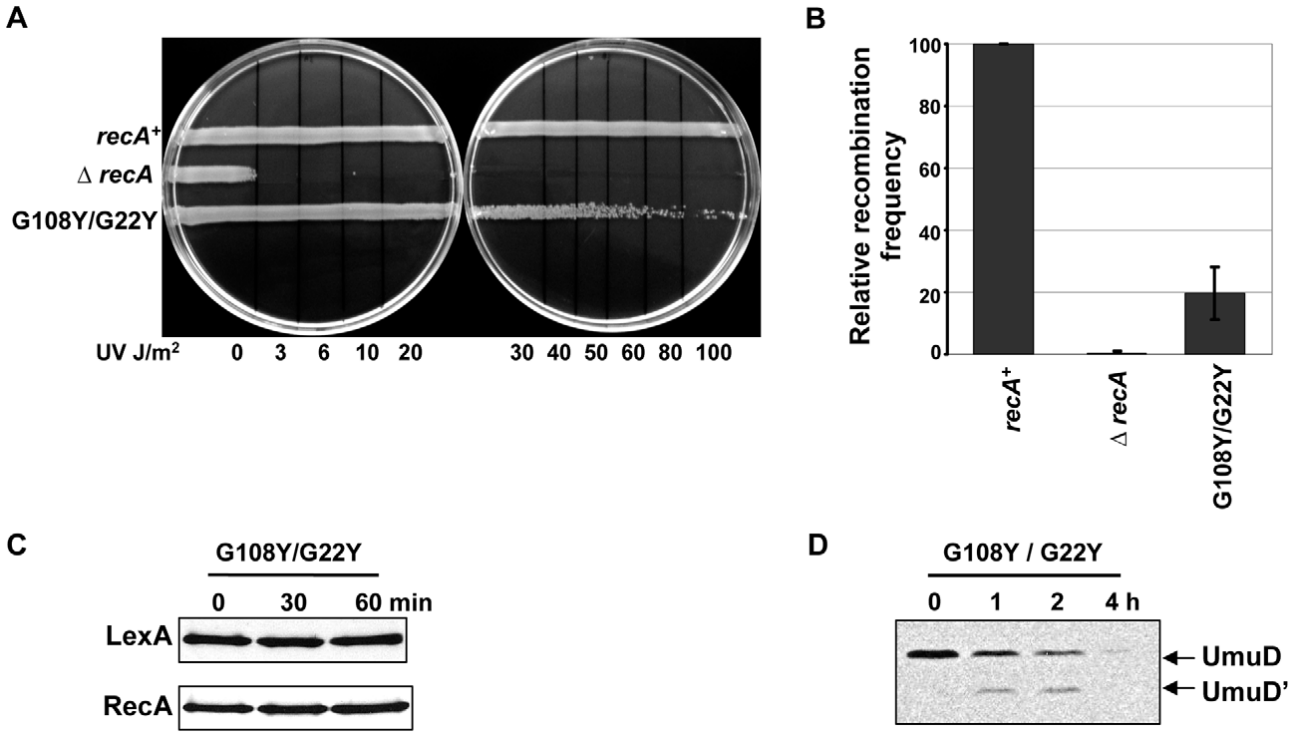
Combined role of G108 and G22 in initiating LexA cleavage. (A) UV survival assay. The double mutant G108Y/G22Y was sensitive to UV damage at dosages 80–100 J/m^2^ respectively. (B) P1 transduction assay showing the double mutant G108Y/G22Y retaining up to 20% as that of *recA*-WT (C) LexA cleavage induction. The double mutant G108Y/G22Y could not induce LexA cleavage as well as no up-regulation of RecA seen, unlike the corresponding single amino acid substitutions. (D) Western analysis of UmuD cleavage. The LexA cleavage deficient *E. coli* strain carrying the RecA G108Y/G22Y double mutation showed UmuD cleavage into UmuD' product, upon DNA damage. All assays were carried out at least 3 times independently, and the representative figures or data representing the mean ± S.E. are shown.

To test the possibility that LexA cleavage induction might require binding RecA at G108 and G22 residues at the same time, we made the double mutant G108Y/G22Y. We reasoned that with both ET-sites 3 and 4 mutated, LexA could not bind to RecA anymore, allowing it to repress the SOS response. In our assays, the double mutant (G108Y/G22Y) was weakly sensitive to UV (Figure 7A) comparable to the *recA430* mutant (G204S) (Figure 6A). The recombination efficiency of the double mutant was intermediate between G108Y and G22Y individual mutants (20% as that of wild-type RecA) (Figure 7B). The mutant also did not induce LexA cleavage (Figure 7C), but could still cleave UmuD to UmuD' although, less efficiently (Figure 7D). Importantly, RecA upregulation was much reduced compared to the individual mutants. This supports our hypothesis that a joint disruption at ET-sites 3 and 4, *via* double mutations at residues G108 and G22, impairs LexA binding and prevents its sequestration to RecA with subsequent upregulation of SOS genes. The similar impact of individual mutations at residues G108 and G22 and the synergy of their coupled mutations suggest that they may play joint roles in both LexA binding and subsequent cleavage.

## Discussion

This work identifies new surface exposed domains of RecA critical for its recombinase and LexA cleavage functions. The discovery of these residues with the Evolutionary Trace (ET) shows that this computational strategy, based on phylogenetically correlated sequence variations, applies equally well here in prokaryotes as previously in eukaryotes, and that it can efficiently identify key functional residues that evaded detection by several past studies on this highly mutagenized protein [52]. Finally, this work validates the functional role of recent crystallographic evidence for RecA-RecA and RecA-DNA interfaces [51], and suggests a model for the RecA filament-LexA interaction.

### Overall RecA function

To confirm past observations at RecA functional sites and also to validate the ET strategy, we targeted for site-directed mutagenesis the top-ranked Evolutionary Trace residues at the interface defined in the active RecA-ssDNA filament structure [51]. This structure has a ∼12 Å shift of the RecA-RecA interface upon ssDNA binding compared to the inactive structure [39], and it now includes additional residues important for filament formation (G165, S172, R176 and G212 and E123, A168). Consistent with both the inactive and active structures, top-ranked ET residues significantly overlapped the RecA-RecA and RecA-DNA dimer interfaces (Figure 1A and 1B) and their mutations prohibit both recombinase and LexA cleavage activities (Figure 3). This is in line with previous mutations of neighboring residues with similar defects in recombinase or co-protease activities [74]–[84], and it establishes a functional role for the residues in the extended RecA-RecA interface observed structurally in the active RecA filament.

A second set of top-ranked ET residues, ET site-1 (D224, R226, K245), was found in the cleft region of RecA and adds details on the determinants of overall RecA function. This cleft is located in between two adjacent RecA monomers and was previously proposed to bind repressors [39], [79], [80], [85]–[87] and dsDNA [88], [89], possibly through several of the positively charged side chains [86], [89]. The ET site-1 overlaps this region and mutations of D224 and R226 eradicated both recombinase activity and SOS functions of RecA. This suggests that, as above, this site also takes part in forming a functionally active filament, possibly as a functional extension of the neighboring extended interface patch. The inactive RecA filament structure (PDB:1u99) [39] shows ET site-1 next to the RecA homodimerization site. In addition, the active filament structure (PDB:3cmt) [51] shows both R226 and D224 residues binding to the previously disordered DNA binding loop 2 (L2). In fact, the binding partner of R226 in L2 is Glu207, which is an absolutely conserved residue among 64 RecA enzymes [90], [91] and does not tolerate any amino acid substitution without some loss of function, as seen from saturation mutagenesis [74]. Thus, the severe impact of R226 and D224 mutations on both recombination and SOS induction is consistent with ET site-1 contributing to the formation of the active filament and, indirectly, to DNA binding.

### Recombination function

Another set of top-ranked ET residues, ET site-2 (N304, Q300 and G288), is located in the RecA C-terminal domain (CTD). The CTD region of RecA has been previously implicated in recombinase function [79], [92]–[94], acting as a secondary DNA (dsDNA) binding pocket on the outer surface of the filament. Mutations of all three ET site-2 residues impair recombinase activity but not LexA cleavage (Figure 5B and 5C). These residues are in the edge of the filament groove, and might provide binding stability to dsDNA for its efficient uptake into the filament. Of note, mutation N304D showed a striking separation of function with a complete destruction of recombinase activity similar to *ΔrecA* strain. Such marked defect in recombinase function was previously reported by a point mutation involving Gly301 to Asp in the CTD [95], [96], suggesting the intolerance of negatively charged amino acid side chains in the RecA CTD in dsDNA binding during the recombination process. Alternatively, these residues might modulate interaction between RecA and DinI [97], RecX [22] or RecFOR proteins.

### RecA–LexA interaction

**Figure 8:**
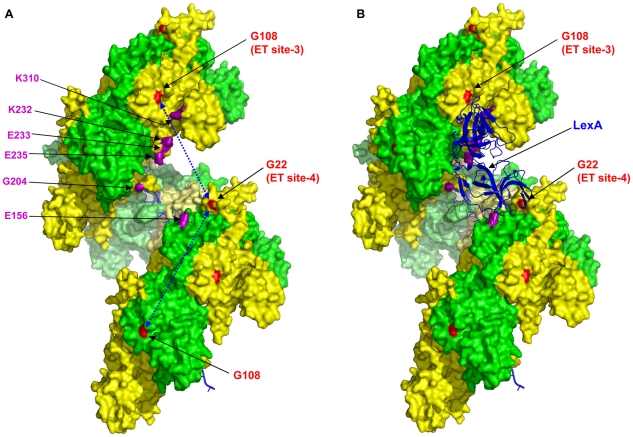
Structure of RecA active filament showing positions of G108 and G22 and residues implicated in LexA cleavage. (A) The crystal structure of RecA active filament (PDB:3cmv) showing positions of G108 and G22 (shaded red) and G204 (shaded magenta) facing the major helical groove. The positions of other residue mutations previously published to have a role on cleavable substrates binding to RecA along the major groove are also shown shaded in magenta color. The possible fits in which LexA can interact with G108 in one RecA monomer and G22 in another RecA monomer across the major groove are shown by double-sided arrows in the left panel (A). *In silico* docking model showing, among other possible solutions, one in which the LexA dimer (blue ribbon structure) docks within 6 Å of residue G108 in one RecA monomer (i) and residue G22 in another RecA monomer (i+6). The LexA model used was a hybrid of the PDB structures 1jhc and 1jhe.

The induction of the SOS response by RecA-mediated cleavage of LexA has been extensively studied both *in vivo* and *in vitro*, yet the sites involved in the interaction of these proteins remain unclear. Our ET analysis reveals two new sites with some potential to be determinants of the RecA-LexA interaction. Mutation of these sites preserves recombination but in majority, inhibits LexA cleavage. Paradoxically, levels of the LexA-repressed proteins, RecA (Figure 6C) and UmuD (data not shown) were up-regulated upon DNA damage; indicating that there was SOS induction, independent of LexA cleavage. These mutants could promote UmuD cleavage; indicating that this defect in co-protease function was highly specific to LexA (Figure 6D). To our knowledge, the activation of the SOS response by UV, independent of LexA cleavage, has not been previously observed. Electron micrograph [20], [98], [99] and mutational studies [71], [72], [79], [80], [82], [83], [85]–[87] point to the binding of LexA, cI and UmuD structural homologs deep within the RecA filament's helical groove. However, structural elements on the edge of the helical groove including the dynamic N-terminal helix/strand (1–30) and C-terminal domain (270–333), have also been found to contribute to cleavage of LexA, cI and UmuD [79], [99]. Consistent with these findings, the residues that we find to be highly specific to LexA hydrolysis lie between the N-terminal α-helix A and β-strand 0 (G22 and K23) or adjacent to the CTD (G108). We propose that LexA binds across the RecA filament's groove through direct contacts at both of the two distant ET-sites 3 and 4, and that these sites cooperate to enable LexA proteolysis (see Figure 8A). Then, as observed, the disruption of either one could permit binding but not cleavage of LexA, leading to SOS induction without efficient LexA degradation. Previous mutational studies [74], [79], [82], [83] implicating residues facing the helical groove in repressor cleavage functions (Figure 8A, shown in magenta) are consistent with this model. Moreover, this model predicts that the simultaneous disruption of both LexA binding sites 3 and 4 would prevent LexA binding and sequestration. Indeed, the G108Y/G22Y double-mutant does not up-regulate expression of RecA (Figure 7C) and UmuD (data not shown). An alternative possibility would be that each point-mutation changes the conformation of the active RecA filament to prevent LexA cleavage. However, such an allosteric effect would have to be subtle since both the recombination function of RecA and its co-protease activity towards UmuD are still present in both the point-mutant and the double-mutant (Figure 7B and 7D). Nevertheless, the less efficient co-protease activity of the double mutant towards UmuD also suggests that the binding sites for UmuD might be shared among these residues or their neighbors, so that the double mutation either directly disrupts the efficiency of UmuD binding and/or cleavage or indirectly affects the protein fold for UmuD binding. This is in agreement with previous electron micrograph and mutational studies suggesting the possibility of repressors sharing similar binding sites on the RecA filament [79], [87], [98].

Consistent with our model of LexA binding to a composite site, a geometric docking analysis of LexA dimer binding to the RecA filament identifies, among many other possible solutions, one in which the LexA dimer binds to ET site-3 and ET site-4 from RecA units at positions *i* and *i*+6, or one helical turn apart, across the filament groove (Figure 8B). This illustrates how, by wedging itself into the groove, the DNA binding domain of LexA may bind the core of the RecA filament and at the same time allow the catalytic C-terminal domain of LexA to span the helical filament's edge. The model could be further addressed by direct assays measuring LexA binding and proteolysis in these mutant proteins *in vitro*. In the future, these RecA mutants may become a useful tool for trapping the RecA-LexA interaction towards efforts to obtain a co-crystal structure. Overall our results suggest that a cooperative binding at RecA residues G108 and G22 is essential for triggering LexA proteolysis.

In conclusion, ET identified new functional sites and efficiently guided their mutational validation in RecA. These sites form important new targets for future biochemical studies of RecA function, and may prove useful for creating separation of function mutants that will help dissect the network of interactions responsible for DNA damage repair. The emergence of bacterial resistance to antibiotics is mediated in part by the SOS response and it has been proposed that blocking the SOS pathway may prevent the evolution of bacteria in contact with these antibiotics [100], [101]. The new RecA sites identified in this work may become useful for the design of new drugs preventing the evolution of bacteria to antibiotics.

## Materials and Methods

### Materials

The low copy plasmid pGE591 containing wild-type *recA* [81] was a kind gift from Dr. George Weinstock, Washington University in St. Louis, MO, and the *E. coli* strain SMR6765 [70] lacking functional RecA was provided by Dr. Susan Rosenberg, Baylor College of Medicine, TX. *E. coli* strain CH458 (*lacZYA*::*gfp-cat*) was used as donor strain for P1 phage lysate preparation. Rabbit anti-UmuDD' polyclonal antiserum [102] was generously provided by Dr. Roger Woodgate (Laboratory of Genomic Integrity, NIH, MD).

### Bacterial strains and plasmids

The genotypes and sources of the *E. coli* strains and plasmids used in this study are listed in Table S1. Strains made in this study were constructed by classical P1 transduction [103].

### Sequence and structure analysis

The Evolutionary Trace analysis [104] used a sequence alignment consisting of 201 RecA protein sequences, nearly all bacterial, that have LexA or LexA homolog (Table S2). The primary source of the alignment was the HSSP database and it was retrieved using the Evolutionary Trace Report Maker Server [105]. Each sequence was BLASTed against the NCBI non-redundant protein sequences (nr) database and the sequences with at most 20 gaps or additions relative to the RecA sequence of *E. coli* were aligned using MUSCLE [106]. This dedicated alignment spanned greater evolutionary distances than the one provided automatically by the ET viewer software [107] (ET servers and viewing tools are available for public use at http://mammoth.bcm.tmc.edu/). The ET phylogenetic tree and multiple sequence alignment of RecA sequences in text and image formats are also available at http://mammoth.bcm.tmc.edu/AdikesavanEtAl/Sup. The interfaces of RecA with ATP, DNA and other monomers were defined as the amino acids that are closer than 5 Å from the ligand in atom to atom distances, excluding hydrogens. The figures of RecA monomer and filament structures were generated by PyMOL (The PyMOL Molecular Graphics System, Version 1.3, Schrödinger, LLC) using the PDB structure: 3cmw. The RecA filament was extended by repeated duplications and space alignment of the terminal monomers. 34045 rigid-body protein-protein docking models of a LexA dimer bound to the RecA filament with good molecular shape complementarity were created with the program PatchDock [108].

### Site-directed mutagenesis of *E. coli* RecA protein

The wild-type RecA plasmid (pGE591-*recA*-WT) was used as template for site-directed mutagenesis of RecA protein using QuikChange II XL site-directed mutagenesis kit (Stratagene) as per manufacturer's protocol. The plasmids containing mutations in the *recA* gene obtained by site-directed mutagenesis were transformed in *E. coli* SMR6765 (Δ*recA*) strain. All the *recA* mutant plasmids were sequence verified. The mutant RecA proteins expressed from these strains were also checked for their stability by western analysis using anti-RecA antibody.

### UV survival assay

The semi-quantitative measurement of UV sensitivities of wild-type RecA and RecA mutants was done as described previously [79]. *E. coli* SMR6765 strains expressing either wild-type RecA or RecA mutants were grown overnight in Luria-Bertani (LB) medium containing selective antibiotic (kanamycin 25 µg/mL). The next day, subcultures were made and grown further till the OD_600_ reached 0.5. The bacterial cultures were streaked onto sterile LB/Kanamycin plates using sterile Q-tips. The plates were exposed to increasing doses (J/m2) of UV light using a UV Stratalinker, and incubated at 37°C for a further period of 16 hours protected from light. Different levels of UV survival between wild-type RecA and RecA mutant strains were analyzed. The assay was repeated at least three times independently and the representative results are shown.

### *In vivo* LexA cleavage analysis by Western blot

Western blot analysis of *in vivo* LexA cleavage was carried out as described previously [79], [109] with minor modifications. *E. coli* SMR6765 strains carrying either wild-type RecA or RecA mutants were grown overnight and the next day, subcultures made and grown at 37°C till the OD_600_ reached 0.5. The DNA damaging agent, nalidixic acid (Sigma) was added to each culture at 100 µg/mL final concentration. The cultures were grown further at 37°C and 1 mL of culture from each strain was aliquoted at 0, 30 and 60 minutes. The culture aliquots were washed once in cold PBS and were stored at −80°C until further processing. Subsequently, the pellets were lysed using BugBuster Master Mix (Novagen) and the total lysate made as per the manufacturer's protocol. Total proteins in the lysates were estimated using the Micro BCA Protein Assay Kit (Thermo Scientific). The RecA protein levels were normalized to bacterial growth by using equal amount (50 µg) of total protein lysate collected at different time points for resolving in SDS-PAGE. The resolved bands were blotted to nitrocellulose membranes and probed with anti-LexA (1∶7000, ABR bioreagents) and anti-RecA (1∶15000, MBL International) antibodies. Goat anti-rabbit IgG-HRP (Chemicon International) was used as the secondary antibody at 1∶7000 dilutions. Chemiluminescence detection was done using Amersham ECL western blotting kit and autoradiographed as per manufacturer's protocol. All the experiments were repeated at least three times for each RecA mutants and the representative results are shown.

### P1 transduction assay

The recombination efficiency of the *E. coli* strains carrying wild-type RecA and RecA mutant proteins were assayed by P1 transduction as described [110]. The assay measures the efficiency of the wild-type RecA or its variants, to recombine the selectable genetic marker (*gfp-cat* gene) into their chromosome, using P1 phage mediated transduction. P1 lysate was prepared by growing the donor bacterial strain (CH458≡MG1655 *lacZYA*::*gfp-cat*) overnight in LB medium with chloramphenicol antibiotic. The overnight culture was diluted 1∶4 in fresh LB+ 5 mM CaCl_2_ and 0.2% glucose and allowed to stand for 30 min at room temperature. Then wild-type P1 phage lysate was added to the diluted overnight culture, incubated with shaking @ 37°C for 20 min followed by plating them on LB plates with 5 mM CaCl_2_ and 0.2% glucose. Next day after overnight incubation of the plates, the top layer of lysed cells were scrapped-off into sterile centrifuge tubes, and ∼300 µl of chloroform added to the lysate, vortexed and allowed to stand for 30 min at room temperature with intermittent vortexing followed by centrifugation @ 10000 rpm for 10 min to collect the supernatant P1 lysate. The P1 phage lysate was subsequently titred against *E. coli* strain SMR6765 containing wild-type RecA on pGE591 plasmid. The viable cell numbers for wild-type RecA and RecA mutant strains was also assayed, so that approximately 1 phage for every 100 viable cells was used in the P1 transduction assay. During the assay, the recipient bacterial strains (wild-type RecA and the RecA-mutant strains) were grown overnight and subcultured the next day till the OD_600_ reached 0.5. P1 lysate was added to the cultures in such a way that the ratio of phage to viable cell count was ∼1∶100, vortexed, and incubated with shaking @ 37°C for 18 min followed by centrifugation for 2 min at 7000 rpm to pellet the cells. The cells were resuspended in LB medium with 100 mM sodium citrate and plated on LB-citrate plates with chloramphenicol, incubated overnight at 37°C. Next day, the number of transductant colonies in each strain was counted. The transduction or recombination efficiency of the wild-type RecA and mutant RecA strains were calculated by the number of transductants relative to the phage titer. The assay was repeated at least 3 times for all the wild-type RecA or RecA mutant strains and the mean standard error values for recombination efficiency were used for graphical representation.

### Analysis of UmuDD' proteins by Western blot

The cleavage of UmuD protein to UmuD' upon DNA damage were shown individually in *E. coli* strains with plasmid-borne wild-type RecA or empty vector or RecA mutants (G108Y, G22Y, K23Y and G24Y) by western blot [111]. The *E. coli* strains (OL53) used in this assay were *lexA* (def) to enable constitutive UmuD expression. UmuD cleavage to UmuD' was assayed similar to LexA cleavage analysis except that after DNA damage induction, the aliquots were collected at 0, 1, 2 and 4 hours (since UmuD induction is a late process in the SOS response). The culture aliquots were processed similarly as mentioned above for LexA cleavage analysis. 200 µg of total protein from lysates were resolved in SDS-PAGE and immunoblotting was done with anti-UmuDD' antisera (1∶2000). The analyses were repeated at least 3 times independently for each wild-type RecA or mutant strains and the representative data were shown.

## Supporting Information

Figure S1 The phylogenetic tree of the RecA sequences. It was generated by the ETC code (http://mammoth.bcm.tmc.edu/downloads.html), using the Unweighted Pair Group Method with Arithmetic Mean (UPGMA). The organism names were obtained from the NCBI entries of the RecA sequences.
(TIF)

Figure S2 The multiple sequence alignment of the RecA protein family. The RecA sequences obtained from the HSSP database for the PDB structure 1u99 were BLASTed against the NCBI non-redundant protein sequences nr database and the sequences with at most 20 gaps or additions relative to the RecA sequence of *E. coli* were aligned using MUSCLE. The graphical illustration was made by using SeaView (http://mac.softpedia.com/get/Math-Scientific/SeaView.shtml). The sequence names were replaced by the organism names according to the NCBI entries.
(TIF)

Figure S3 Mitomycin C survival assay of selected RecA mutant strains. Overnight grown cultures of wild-type *recA*, Δ*recA* and *recA* mutant strains were subcultured the next day and their OD_600_ were adjusted to 0.2. The bacterial cultures were streaked onto LB agar plates carrying a concentration gradient of mitomycin C across the plate. The mitomycin C gradient plates were made by pouring 25 mL of LB agar with 0.8 µg/mL of mitomycin C on a 150 mm petri plate and the plates were lifted at one end to create an agar slant when the agar gets solidified. Once the first layer hardens, LB agar without mitomycin C was poured over the slant to make a flat surface on the top, thus creating a mitomycin concentration gradient across the plate. The sensitivity of each bacterial strain streaked on the LB agar was analyzed qualitatively. The mutants E154A (RecA-RecA/DNA *interface patch*), D224A (ET site-1), N304D (ET site-2) were sensitive to the drug [very faint bacterial growth seen at the low mitomycin C concentration region of the agar plate], while T89A (bottom-ranked ET residue), G108Y (ET site-3), G22Y (ET site-4) and the double mutant G108Y/G22Y were resistant [bacterial growth seen up to half of the plate]. The mitomycin C survival phenotypes of the bacterial strains checked were comparable to their UV sensitivities indicating that the phenotypes observed were not UV-specific.
(TIF)

Table S1 *Escherichia coli* K12 strains and plasmids used.
(DOC)

Table S2 List of RecA protein sequences used for the ET analysis. The sequence names were replaced by the organism names according to the NCBI entries.
(DOCX)
