# Supplementary figures and images for: Separation of Recombination and SOS Response in Escherichia coli RecA Suggests LexA Interaction Sites
Source: PLoS Genet. 2011 Sep 1;7(9):e1002244. doi: 10.1371/journal.pgen.1002244 (PMC3164682; doi:10.1371/journal.pgen.1002244)

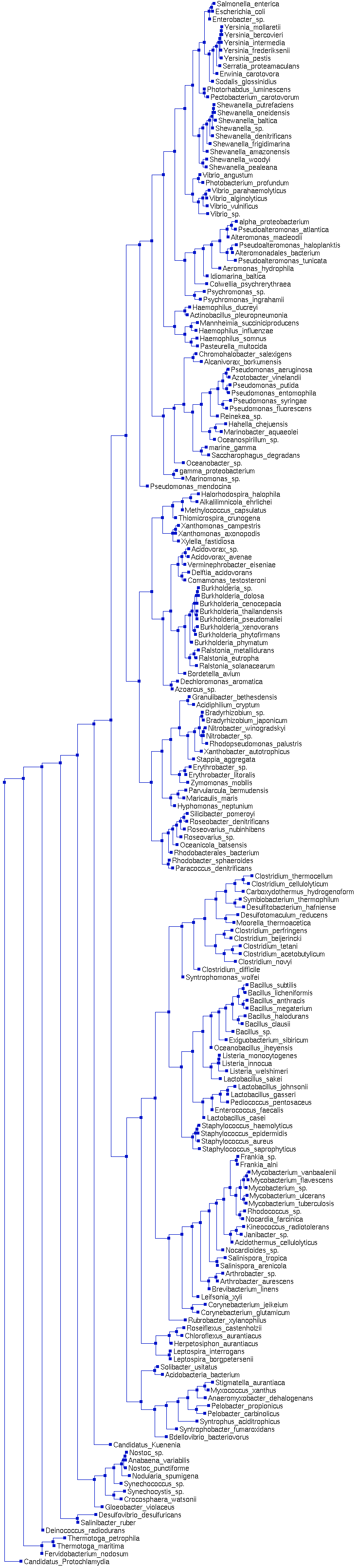

Supplement: Figure S1 — The phylogenetic tree of the RecA sequences. It was generated by the ETC code (http://mammoth.bcm.tmc.edu/downloads.html), using the Unweighted Pair Group Method with Arithmetic Mean (UPGMA). The organism names were obtained from the NCBI entries of the RecA sequences. (TIF) [file pgen.1002244.s001.tif]

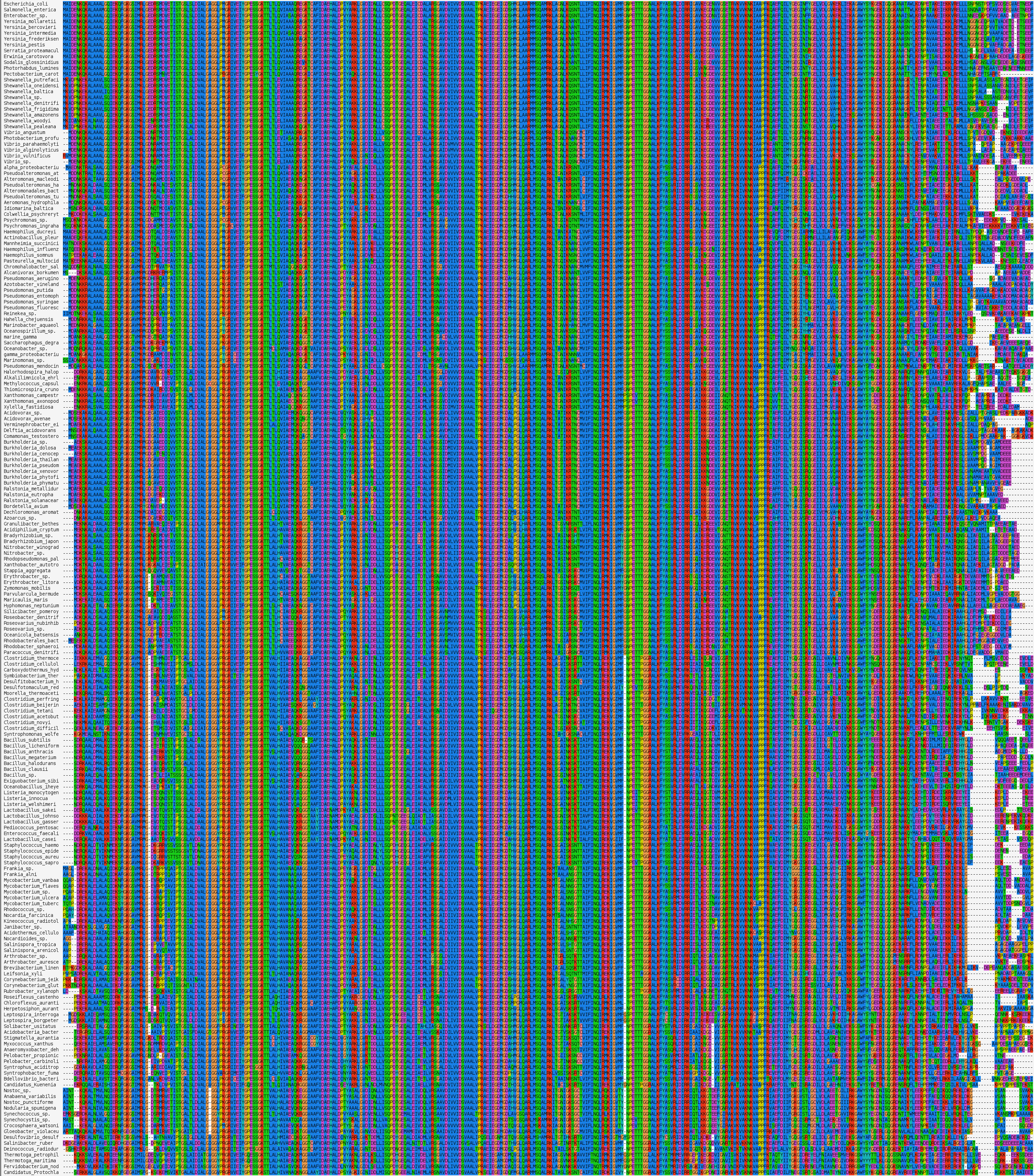

Supplement: Figure S2 — The multiple sequence alignment of the RecA protein family. The RecA sequences obtained from the HSSP database for the PDB structure 1u99 were BLASTed against the NCBI non-redundant protein sequences nr database and the sequences with at most 20 gaps or additions relative to the RecA sequence of E. coli were aligned using MUSCLE. The graphical illustration was made by using SeaView (http://mac.softpedia.com/get/Math-Scientific/SeaView.shtml). The sequence names were replaced by the organism names according to the NCBI entries. (TIF) [file pgen.1002244.s002.tif]

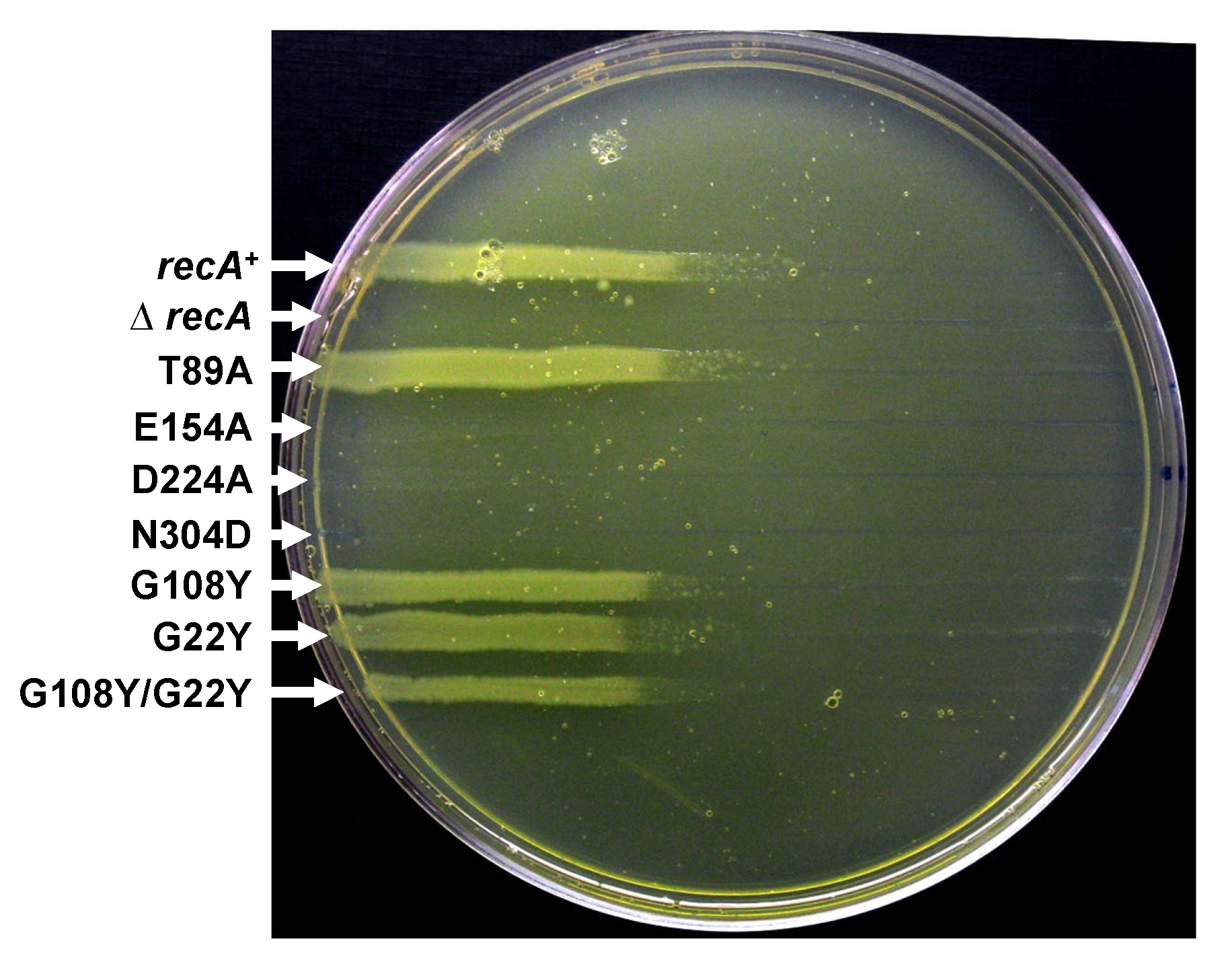

Supplement: Figure S3 — Mitomycin C survival assay of selected RecA mutant strains. Overnight grown cultures of wild-type recA, ΔrecA and recA mutant strains were subcultured the next day and their OD600 were adjusted to 0.2. The bacterial cultures were streaked onto LB agar plates carrying a concentration gradient of mitomycin C across the plate. The mitomycin C gradient plates were made by pouring 25 mL of LB agar with 0.8 µg/mL of mitomycin C on a 150 mm petri plate and the plates were lifted at one end to create an agar slant when the agar gets solidified. Once the first layer hardens, LB agar without mitomycin C was poured over the slant to make a flat surface on the top, thus creating a mitomycin concentration gradient across the plate. The sensitivity of each bacterial strain streaked on the LB agar was analyzed qualitatively. The mutants E154A (RecA-RecA/DNA interface patch), D224A (ET site-1), N304D (ET site-2) were sensitive to the drug [very faint bacterial growth seen at the low mitomycin C concentration region of the agar plate], while T89A (bottom-ranked ET residue), G108Y (ET site-3), G22Y (ET site-4) and the double mutant G108Y/G22Y were resistant [bacterial growth seen up to half of the plate]. The mitomycin C survival phenotypes of the bacterial strains checked were comparable to their UV sensitivities indicating that the phenotypes observed were not UV-specific. (TIF) [file pgen.1002244.s003.tif]
