# Supplementary material for: Separation of Recombination and SOS Response in Escherichia coli RecA Suggests LexA Interaction Sites
Source: PLoS Genet. 2011 Sep 1;7(9):e1002244. doi: 10.1371/journal.pgen.1002244 (PMC3164682; doi:10.1371/journal.pgen.1002244)
Supplement: Table S1 — Escherichia coli K12 strains and plasmids used. (DOC) [file pgen.1002244.s004.doc]

**Table S1. *Escherichia coli* K12 strains and plasmids**

| Plasmids / Strains | Genotype or relevant genotype | Reference / Source |
| --- | --- | --- |
| MG1655 | F^-^1^-^ *ilvG rfb-50 rph-1*, sequenced wild-type *E. coli* K12 | Laboratory stock |
| pGE523 | *oriF kan* | [1] |
| pGE591 | pGE523 *recA*^+^ | [1] |
| SMR6765 | MG1655 ∆*att*λ::P*_sulA_*Ω*gfp-mut2* ∆(*srlR-recA*)*306*::Tn10 | [2] |
| OL10 | SMR6765 pGE591*-*N181A | This work |
| OL11 | SMR6765 pGE591*-*E123A | This work |
| OL12 | SMR6765 pGE591*-*E154A | This work |
| OL13 | SMR6765 pGE591*-*L126A | This work |
| OL15 | SMR6765 pGE591*-* R226A | This work |
| OL16 | SMR6765 pGE591*-*K245A | This work |
| OL18 | SMR6765 pGE591*-*R176A | This work |
| OL19 | SMR6765 pGE591*-*Q173A | This work |
| OL20 | SMR6765 pGE591*-*G212Y | This work |
| OL21 | SMR6765 pGE591*-*G165W | This work |
| OL24 | SMR6765 pGE591*-*T89A | This work |
| OL25 | SMR6765 pGE591*-*N186A | This work |
| OL26 | SMR6765 pGE591*-*V238A | This work |
| OL27 | SMR6765 pGE591*-*K294A | This work |
| OL28 | SMR6765 pGE591*-*N312A | This work |
| OL29 | SMR6765 pGE591*-*S172W | This work |
| OL31 | SMR6765 pGE591*-*D224A | This work |
| OL33 | SMR6765 pGE591*-*N304D | This work |
| OL34 | SMR6765 pGE591*-*G288Y | This work |
| OL35 | SMR6765 pGE591*-*Q300A | This work |
| OL36 | SMR6765 pGE591*-*G108Y | This work |
| OL37 | SMR6765 pGE591*-*G87Y | This work |
| OL38 | SMR6765 pGE591*-*K88Y | This work |
| OL39 | SMR6765 pGE591*-*G22Y | This work |
| OL40 | SMR6765 pGE591*-*K23Y | This work |
| OL41 | SMR6765 pGE591*-*G24Y | This work |
| OL47 | SMR6765 pGE591*-*G108Y-G22Y | This work |
| OL51 | SMR6765 pGE591*-*A168Y | This work |
| OL52 | SMR6765 pGE591*-*G204S | This work |
| SMR7623 | MG1655 Δ*att*λ:: P*_sulA_*Ω*gfp-mut2* *lexA*51 *sulA*211 *malB*::Tn9 | [2] |
| OL53 | MG1655 Δ*att*λ:: P*_sulA_*Ω*gfp-mut2* *lexA*51 *sulA*211 | SMR7623 X P1 (MG1655)  This work |
| GY8322 | *supE44 Δ(srlR-recA)306::Tn*10 *thr-1 leuB6 Δ(gpt-proA) hisG4 argE3 thi-1 araC14 lacY1galK2 xylA5 mtl-1 sfiA11 tsx-33 qsr' rfbD1 mgl-51 rpsL31 kdgK51* [K5353=mini-F *recA^+^*] *λ-* | [3] |
| OL54 | MG1655 Δ*att*λ:: P*_sulA_*Ω*gfp-mut2* *lexA*51 *sulA*211 ∆(*srlR-recA*) *306*::Tn10 | OL53 X P1 (GY8322)  This work |
| OL60 | OL54 pGE591 | This work |
| OL61 | OL54 pGE591*-*G108Y | This work |
| OL62 | OL54 pGE591*-*G22Y | This work |
| OL63 | OL54 pGE591*-*K23Y | This work |
| OL64 | OL54 pGE591*-*G24Y | This work |
| OL65 | OL54 pGE591*-*G204S | This work |
| OL66 | OL54 pGE591*-*G108Y-G22Y | This work |
| CH458 | MG1655 *lacZYA*::*gfp-cat* | [4] |

1. Weisemann JM, Weinstock GM (1988) Mutations at the cysteine codons of the recA gene of Escherichia coli. DNA 7: 389-398.

2. Pennington JM, Rosenberg SM (2007) Spontaneous DNA breakage in single living Escherichia coli cells. Nat Genet 39: 797-802.

3. Asai T, Sommer S, Bailone A, Kogoma T (1993) Homologous recombination-dependent initiation of DNA replication from DNA damage-inducible origins in Escherichia coli. Embo J 12: 3287-3295.

4. Gordon AJ, Halliday JA, Blankschien MD, Burns PA, Yatagai F, et al. (2009) Transcriptional infidelity promotes heritable phenotypic change in a bistable gene network. PLoS Biol 7: e44.
