# Supplementary material for: Separation of Recombination and SOS Response in Escherichia coli RecA Suggests LexA Interaction Sites
Source: PLoS Genet. 2011 Sep 1;7(9):e1002244. doi: 10.1371/journal.pgen.1002244 (PMC3164682; doi:10.1371/journal.pgen.1002244)
Supplement: Table S2 — List of RecA protein sequences used for the ET analysis. The sequence names were replaced by the organism names according to the NCBI entries. (DOCX) [file pgen.1002244.s005.docx]

**Table S2. List of RecA protein sequences used for ET analysis (**The sequence names were replaced by the organism names according to the NCBI entries).

| *Escherichia_coli* |
| --- |
| *Salmonella_enterica* |
| *Enterobacter_sp.* |
| *Yersinia_mollaretii* |
| *Yersinia_bercovieri* |
| *Yersinia_intermedia* |
| *Yersinia_frederiksenii* |
| *Yersinia_pestis* |
| *Serratia_proteamaculans* |
| *Erwinia_carotovora* |
| *Sodalis_glossinidius* |
| *Photorhabdus_luminescens* |
| *Pectobacterium_carotovorum* |
| *Shewanella_putrefaciens* |
| *Shewanella_oneidensis* |
| *Shewanella_baltica* |
| *Shewanella_sp.* |
| *Shewanella_denitrificans* |
| *Shewanella_frigidimarina* |
| *Shewanella_amazonensis* |
| *Shewanella_woodyi* |
| *Shewanella_pealeana* |
| *Vibrio_angustum* |
| *Photobacterium_profundum* |
| *Vibrio_parahaemolyticus* |
| *Vibrio_alginolyticus* |
| *Vibrio_vulnificus* |
| *Vibrio_sp.* |
| *alpha_proteobacterium* |
| *Pseudoalteromonas_atlantica* |
| *Alteromonas_macleodii* |
| *Pseudoalteromonas_haloplanktis* |
| *Alteromonadales_bacterium* |
| *Pseudoalteromonas_tunicata* |
| *Aeromonas_hydrophila* |
| *Idiomarina_baltica* |
| *Colwellia_psychrerythraea* |
| *Psychromonas_sp.* |
| *Psychromonas_ingrahamii* |
| *Haemophilus_ducreyi* |
| *Actinobacillus_pleuropneumonia* |
| *Mannheimia_succiniciproducens* |
| *Haemophilus_influenzae* |
| *Haemophilus_somnus* |
| *Pasteurella_multocida* |
| *Chromohalobacter_salexigens* |
| *Alcanivorax_borkumensis* |
| *Pseudomonas_aeruginosa* |
| *Azotobacter_vinelandii* |
| *Pseudomonas_putida* |
| *Pseudomonas_entomophila* |
| *Pseudomonas_syringae* |
| *Pseudomonas_fluorescens* |
| *Reinekea_sp.* |
| *Hahella_chejuensis* |
| *Marinobacter_aquaeolei* |
| *Oceanospirillum_sp.* |
| *marine_gamma* |
| *Saccharophagus_degradans* |
| *Oceanobacter_sp.* |
| *gamma_proteobacterium* |
| *Marinomonas_sp.* |
| *Pseudomonas_mendocina* |
| *Halorhodospira_halophila* |
| *Alkalilimnicola_ehrlichei* |
| *Methylococcus_capsulatus* |
| *Thiomicrospira_crunogena* |
| *Xanthomonas_campestris* |
| *Xanthomonas_axonopodis* |
| *Xylella_fastidiosa* |
| *Acidovorax_sp.* |
| *Acidovorax_avenae* |
| *Verminephrobacter_eiseniae* |
| *Delftia_acidovorans* |
| *Comamonas_testosteroni* |
| *Burkholderia_sp.* |
| *Burkholderia_dolosa* |
| *Burkholderia_cenocepacia* |
| *Burkholderia_thailandensis* |
| *Burkholderia_pseudomallei* |
| *Burkholderia_xenovorans* |
| *Burkholderia_phytofirmans* |
| *Burkholderia_phymatum* |
| *Ralstonia_metallidurans* |
| *Ralstonia_eutropha* |
| *Ralstonia_solanacearum* |
| *Bordetella_avium* |
| *Dechloromonas_aromatica* |
| *Azoarcus_sp.* |
| *Granulibacter_bethesdensis* |
| *Acidiphilium_cryptum* |
| *Bradyrhizobium_sp.* |
| *Bradyrhizobium_japonicum* |
| *Nitrobacter_winogradskyi* |
| *Nitrobacter_sp.* |
| *Rhodopseudomonas_palustris* |
| *Xanthobacter_autotrophicus* |
| *Stappia_aggregata* |
| *Erythrobacter_sp.* |
| *Erythrobacter_litoralis* |
| *Zymomonas_mobilis* |
| *Parvularcula_bermudensis* |
| *Maricaulis_maris* |
| *Hyphomonas_neptunium* |
| *Silicibacter_pomeroyi* |
| *Roseobacter_denitrificans* |
| *Roseovarius_nubinhibens* |
| *Roseovarius_sp.* |
| *Oceanicola_batsensis* |
| *Rhodobacterales_bacterium* |
| *Rhodobacter_sphaeroides* |
| *Paracoccus_denitrificans* |
| *Clostridium_thermocellum* |
| *Clostridium_cellulolyticum* |
| *Carboxydothermus_hydrogenoform* |
| *Symbiobacterium_thermophilum* |
| *Desulfitobacterium_hafniense* |
| *Desulfotomaculum_reducens* |
| *Moorella_thermoacetica* |
| *Clostridium_perfringens* |
| *Clostridium_beijerincki* |
| *Clostridium_tetani* |
| *Clostridium_acetobutylicum* |
| *Clostridium_novyi* |
| *Clostridium_difficile* |
| *Syntrophomonas_wolfei* |
| *Bacillus_subtilis* |
| *Bacillus_licheniformis* |
| *Bacillus_anthracis* |
| *Bacillus_megaterium* |
| *Bacillus_halodurans* |
| *Bacillus_clausii* |
| *Bacillus_sp.* |
| *Exiguobacterium_sibiricum* |
| *Oceanobacillus_iheyensis* |
| *Listeria_monocytogenes* |
| *Listeria_innocua* |
| *Listeria_welshimeri* |
| *Lactobacillus_sakei* |
| *Lactobacillus_johnsonii* |
| *Lactobacillus_gasseri* |
| *Pediococcus_pentosaceus* |
| *Enterococcus_faecalis* |
| *Lactobacillus_casei* |
| *Staphylococcus_haemolyticus* |
| *Staphylococcus_epidermidis* |
| *Staphylococcus_aureus* |
| *Staphylococcus_saprophyticus* |
| *Frankia_sp.* |
| *Frankia_alni* |
| *Mycobacterium_vanbaalenii* |
| *Mycobacterium_flavescens* |
| *Mycobacterium_sp.* |
| *Mycobacterium_ulcerans* |
| *Mycobacterium_tuberculosis* |
| *Rhodococcus_sp.* |
| *Nocardia_farcinica* |
| *Kineococcus_radiotolerans* |
| *Janibacter_sp.* |
| *Acidothermus_cellulolyticus* |
| *Nocardioides_sp.* |
| *Salinispora_tropica* |
| *Salinispora_arenicola* |
| *Arthrobacter_sp.* |
| *Arthrobacter_aurescens* |
| *Brevibacterium_linens* |
| *Leifsonia_xyli* |
| *Corynebacterium_jeikeium* |
| *Corynebacterium_glutamicum* |
| *Rubrobacter_xylanophilus* |
| *Roseiflexus_castenholzii* |
| *Chloroflexus_aurantiacus* |
| *Herpetosiphon_aurantiacus* |
| *Leptospira_interrogans* |
| *Leptospira_borgpetersenii* |
| *Solibacter_usitatus* |
| *Acidobacteria_bacterium* |
| *Stigmatella_aurantiaca* |
| *Myxococcus_xanthus* |
| *Anaeromyxobacter_dehalogenans* |
| *Pelobacter_propionicus* |
| *Pelobacter_carbinolicus* |
| *Syntrophus_aciditrophicus* |
| *Syntrophobacter_fumaroxidans* |
| *Bdellovibrio_bacteriovorus* |
| *Candidatus_Kuenenia* |
| *Nostoc_sp.* |
| *Anabaena_variabilis* |
| *Nostoc_punctiforme* |
| *Nodularia_spumigena* |
| *Synechococcus_sp.* |
| *Synechocystis_sp.* |
| *Crocosphaera_watsonii* |
| *Gloeobacter_violaceus* |
| *Desulfovibrio_desulfuricans* |
| *Salinibacter_ruber* |
| *Deinococcus_radiodurans* |
| *Thermotoga_petrophila* |
| *Thermotoga_maritima* |
| *Fervidobacterium_nodosum* |
| *Candidatus_Protochlamydia* |
